# Exploring comprehensive sexuality education experiences and barriers among students, teachers and principals in Nepal: a qualitative study

**DOI:** 10.1186/s12978-024-01876-0

**Published:** 2024-09-11

**Authors:** Sara Rivenes Lafontan, Felicia Jones, Niru Lama

**Affiliations:** 1 Institute of Nursing and Health Promotion, Faculty of Health Sciences, OsloMet-Oslo Metropolitan University, Oslo, Norway; 2 United Nations Population Fund, UNFPA, Kabul, Afghanistan; 3 University of Oslo, Oslo, Norway

**Keywords:** Comprehensive sexuality education, Adolescents, Sexual health, School, Nepal

## Abstract

**Background:**

Comprehensive sexuality education (CSE) is essential in empowering adolescents with the knowledge and confidence to manage their sexual and reproductive health. Despite its recognized benefits, access to quality CSE remains limited, especially in low-income countries, where societal norms and structural barriers hinder effective delivery. The aim of this study is to investigate the experiences and perceptions among students, teachers, and principals in Nepal about comprehensive sexuality education.

**Methods:**

Qualitative methods were used. 13 Semi-structured interviews and 1 focus group discussion were carried out with 15 teachers and principals working at higher secondary schools and two focus group discussions were conducted with a total of 13 adolescents. Thematic analysis was used to analyze the data.

**Results:**

Four themes were developed: Resistance to Teaching and Learning, Preparation and Engagement Strategies, Taboos and Silencing and Structural Barriers. Students, teachers, principals and students reported discomfort and embarrassment when discussing sensitive topics, with gender dynamics playing a significant role. Strategies like warm-up sessions and continuous interaction with students and parents were used to create a supportive learning environment. However, socio-cultural barriers and family attitudes continued to hinder open discussions about sexuality. Structural barriers, including the lack of formal training for teachers and inadequate instructional materials, further impeded effective CSE delivery.

**Conclusion:**

The experiences of CSE in Nepal among students, teachers and principals highlight significant barriers including cultural taboos, gender dynamics and insufficient resources. Addressing these barriers through comprehensive teacher training, curriculum reform, and societal engagement is critical to ensure access to CSE.

**Supplementary Information:**

The online version contains supplementary material available at 10.1186/s12978-024-01876-0.

## Background

In a world with more than 1.8 billion young people between the ages of 10–24 and 1.9 billion expected to turn 15 between 2015 and 2030, it is critical that the health and well-being of adolescents and young people remain a priority for communities, societies, and governments [1]. This demographic transition demands immediate and sustained efforts to ensure that this large cohort of youth receives the necessary support to lead healthy and fulfilling lives. Global consensus emphasizes the importance of comprehensive approaches to adolescent well-being, considering various factors like health, education, social connectedness, and resilience. Investments in adolescent well-being yield multiple benefits, contributing to health, economic growth, and human capital development [2, 3]. As a cross-cutting theme, adolescent sexual- and reproductive health and rights (ASRHR) is fundamental to prepare young people to fully exercise their inherent human rights, responsibilities, and dignity. However, lack of access to contraceptives, unintended pregnancies, unsafe abortion, early marriage, and gender-based violence, including female genital mutilation, prevent adolescents from exercising these rights [2].

Access to essential health information and services is vital for young individuals to exercise their sexual and reproductive health rights. However, despite increased awareness of their needs, most adolescents still lack access to adequate information and services [4, 5]. Comprehensive sexuality education (CSE) plays a vital role in empowering young individuals to navigate their sexual and reproductive health (SRH) with knowledge and confidence. A curriculum-based process, CSE is designed to teach children and young people about the cognitive, emotional, physical, and social aspects of sexuality. CSE goes beyond simply providing information about sexual health; it aims to equip young individuals with the knowledge, skills, attitudes, and values needed to make informed decisions, develop respectful relationships, and understand and protect their rights throughout their lives. The education is delivered in both formal and non-formal settings and is scientifically accurate, age-appropriate, and culturally relevant. It addresses a wide range of topics, including sexual and reproductive health, human rights, gender equality, and violence prevention, while also promoting critical thinking, self-efficacy, and empathy. By fostering these competencies, CSE contributes to the development of a fair and compassionate society, empowering young people to make responsible decisions and build healthy, respectful relationships [6].

Despite the clear benefits of CSE, many young people globally currently lack access to quality CSE, particularly in low-income countries [4, 5, 7]. Societal norms often discourage open discourse about sexuality, exacerbating challenges like gender-based violence, early marriage, and limited access to contraception. However, significant gaps persist in the implementation of CSE, including limited teacher training and discomfort in addressing sensitive topics. This deficiency in high-quality, developmentally appropriate sexuality education leaves young individuals vulnerable to harmful behaviors and exploitation [6]. Lack of access to CSE highlights a critical need for interventions that are both culturally sensitive and scientifically accurate.

In Nepal, over 25% of the population are between 10 and 19 years old [8]. A significant proportion of the adolescent population, particularly females, are married at a young age, with 56.6% of females aged 16–25 being married, compared to 30.1% of males within the same age bracket. High rates of early marriage, coupled with societal pressures to conceive shortly after, are intrinsically linked to Nepal's economic underdevelopment [9]. Fifty-two per cent of adolescent girls are mothers by the age of 20 [10]. Contraceptive prevalence among married girls aged 15–19 remains low, with only 15% utilizing modern contraceptive methods, while approximately 40% have an unmet need for contraception [9, 11, 12]. Even with a progressive abortion law in place, less than half of all abortions in Nepal are conducted legally in government-approved facilities. The majority are clandestine and unsafe procedures administered by untrained or unapproved providers or performed by the pregnant women themselves [13]. In addition, adolescent girls in Nepal encounter multifaceted challenges such as gender-based violence, menstrual banishment and restrictions, and limited economic opportunities, all of which impact their sexual and reproductive health (SRH) outcomes [8, 14–18].

In addressing the complex landscape of adolescent sexual and reproductive health (ASRH), Nepal has embarked on various initiatives. These include the integration of adolescent-friendly services at primary healthcare facilities, coupled with community-based programs led by Female Community Health Volunteers [19] and implementation of the National Adolescent Sexual and Reproductive Health Programme, a catalysator for adolescent-friendly SRH services [9]. Additionally, sexuality education has been integrated into the national school curriculum aiming to equip adolescents with life skills to navigate critical issues such as puberty, early marriage, and pregnancy [6, 20, 21]. However, despite these efforts, less than 25% of secondary schools in Nepal have fully implemented the national CSE policy [22]. Existing studies highlight socio-cultural norms and taboos surrounding sexual health that continue to impede open discourse and hinder adolescents from accessing necessary support and information [23–25]. This lack of implementation and persistence of socio-cultural barriers make it evident that CSE in Nepal still faces major challenges that require immediate attention.

While efforts have been made to improve the provision of sexuality education in Nepal, there is limited understanding of the specific barriers faced by students, teachers and principals that hinder the effective delivery of CSE. The aim of this study is to investigate the experiences and perceptions among students, teachers, and principals in Nepal about comprehensive sexuality education. The study seeks to understand the barriers students face in accessing CSE, both individually and within their socio-cultural context, as well as the structural challenges within the educational system.

## Methods

A qualitative exploratory approach was chosen to explore the experiences and perceptions of students, teachers, and principals in Nepal concerning comprehensive sexuality education. This method is particularly suited to enhance knowledge about intricate socio-cultural and structural barriers that affect the delivery and understanding of CSE, aligned with the study's aim of capturing the depth and complexity of these challenges [26].

### Study setting

Data was collected in and around the Kathmandu Valley, the educational center of Nepal in 2022. The valley consists of Kathmandu district (the capital city of Nepal), Bhaktapur district and Lalitpur district. The combined population of these districts is approximately 3.5 million, with a diverse mix of urban and semi-urban communities [22]. The Kathmandu Valley hosts over 1000 secondary schools, including a broad spectrum of public, private, and community-run institutions [27]. This diversity in educational settings offers a robust backdrop for examining the implementation of comprehensive sexuality education (CSE), capturing the varied socio-economic and cultural influences that shape educational practices in Nepal. The Valley's schools range from highly resourced private institutions to under-resourced public schools [28].

### Sampling and recruitment

Secondary schools in the Kathmandu Valley were randomly selected and directly contacted by the last author by email with request to participate in the study. Purposeful sampling was used, intentionally selecting participants based on their characteristics, knowledge, experiences relevant to the study aim [26]. Inclusion criteria included speaking Nepalese, teaching Environment Health and Population for ninth or tenth grades or both grades in the school of Kathmandu Valley or working as a principal of secondary or higher secondary schools of Kathmandu Valley. The inclusion criteria for the students were that they were students in Environment Health and Population in ninth or tenth grades in the school of Kathmandu Valley and that could communicate in Nepali. Participants with varying years of teaching experience were intentionally recruited for the study as well as participants with both genders and different age groups with the aim of providing rich, detailed, and diverse perspectives on the topic under investigation [29]. All the potential participants who were contacted agreed to participate in the study. The nine schoolteachers and six principals working at nine higher secondary schools in the Kathmandu Valley agreed to participate. In addition, six boys and seven girls studying at two different schools in the Kathmandu Valley were selected by their teacher for focus group discussion (FGD). After receiving information about the implications of participating in the study from the last author, the teachers and principals signed informed consent forms. The students, as minors, received the same information and their teacher consented on their behalf to participate in the study.

### Data collection

Data was collected between August and December 2022. Semi-structured interviews were chosen as data collection method allowing for exploration of participants' experiences and perceptions by a set of pre-determined open questions, while providing flexibility to probe further into specific topics as needed [29]. Focus group discussions were selected as an additional data collection method to capture a broader range of perspectives and stimulate interactive dialogue among participants, which can reveal collective views and social dynamics thus providing a more comprehensive understanding of the topic of study [30].

In total, 13 semi-structured interviews lasting 30–40 min each were conducted with 13 schoolteachers at 7 different schools and 1 FGD lasting 30–40 min each were conducted among 6 schoolteachers and principals [4 had also participated in semi-structured interviews]. Two FGDs were carried out among students, one with 7 girls and one with 6 boys. The interviews and FGDs were conducted in private rooms within the schools, away from the usual classroom settings and administrative offices, to avoid interruptions and to ensure that participants could speak freely without concerns of being overheard by colleagues, students, or other school staff. See Table 1 for information about the participants and data collection methods. Separate interview guides were used for the semi-structured interviews and FDGs with the teachers, principals, and students (Additional file 1). The interview guides used focused on the experiences of barriers to comprehensive sexuality education. The interview and focus group guides for teachers, principals, and students focused on exploring the inclusion, effectiveness, and delivery methods of CSE in the school curriculum, while also identifying barriers and potential strategies for improvement. They included questions about participants' past and present experiences with CSE, their perceptions of the curriculum, the challenges they face, and suggestions for overcoming these challenges to enhance CSE delivery and effectiveness in Nepal. The interviews were conducted in Nepali by two female research assistants who are both health professionals and who were trained on interview techniques and using the interview guide. The interviews were recorded and transcribed verbatim by the last author.

**Table 1:** Participants and data collection methods

Participants	No. of participants	In-depth Interview (IDI)s conducted	Focus group discussion (FGD) conducted
Students	13		2
Schoolteachers and principals	15	13	1 (among 6 participants, 4 were repeated from IDIs)
Total	26		

### Data analysis

Data were analyzed using Braun and Clarke’s six-step approach to reflexive thematic analysis [31, 32]. The first step included familiarization with the data by listening actively to each interview. In the second phase, meaning units were identified and coded using NVivo software [33] by the last author. The third phase involved sorting and condensing the codes into different themes by reviewing codes. The fourth phase restructured and adjusted the various potential themes and sub-themes. In the fifth phase, the codes are recontextualized into the final themes and in the last phase, the present manuscript was developed. The development of preliminary and final themes was the result of discussions among all authors.

### Ethics

The study was approved by Norwegian Centre for Research Data (Sikt) (ID no:670337) and the Ethical Review Board of Nepal Health Research Council (ERB Protocol Registration No. 801/2020). Written informed consent was obtained from the study participants and from the teachers on behalf of the students. We used an app connected to services for sensitive research data to record the interviews (Nettskjema-University of Oslo). Data was de-identified and anonymized when transcribed. To ensure anonymity, all participants were assigned unique codes, and identifying information was removed from transcripts and reports. Data was stored in encrypted files, accessible only to the research team, and all physical copies of notes were securely locked. The recordings were stored on a secure, password-protected server, and transcriptions were de-identified before analysis. Due to the sensitivity of the topic and the small number of participants involved in the study, details of them are not provided to protect their anonymity.

## Results

**Fig. 1:**
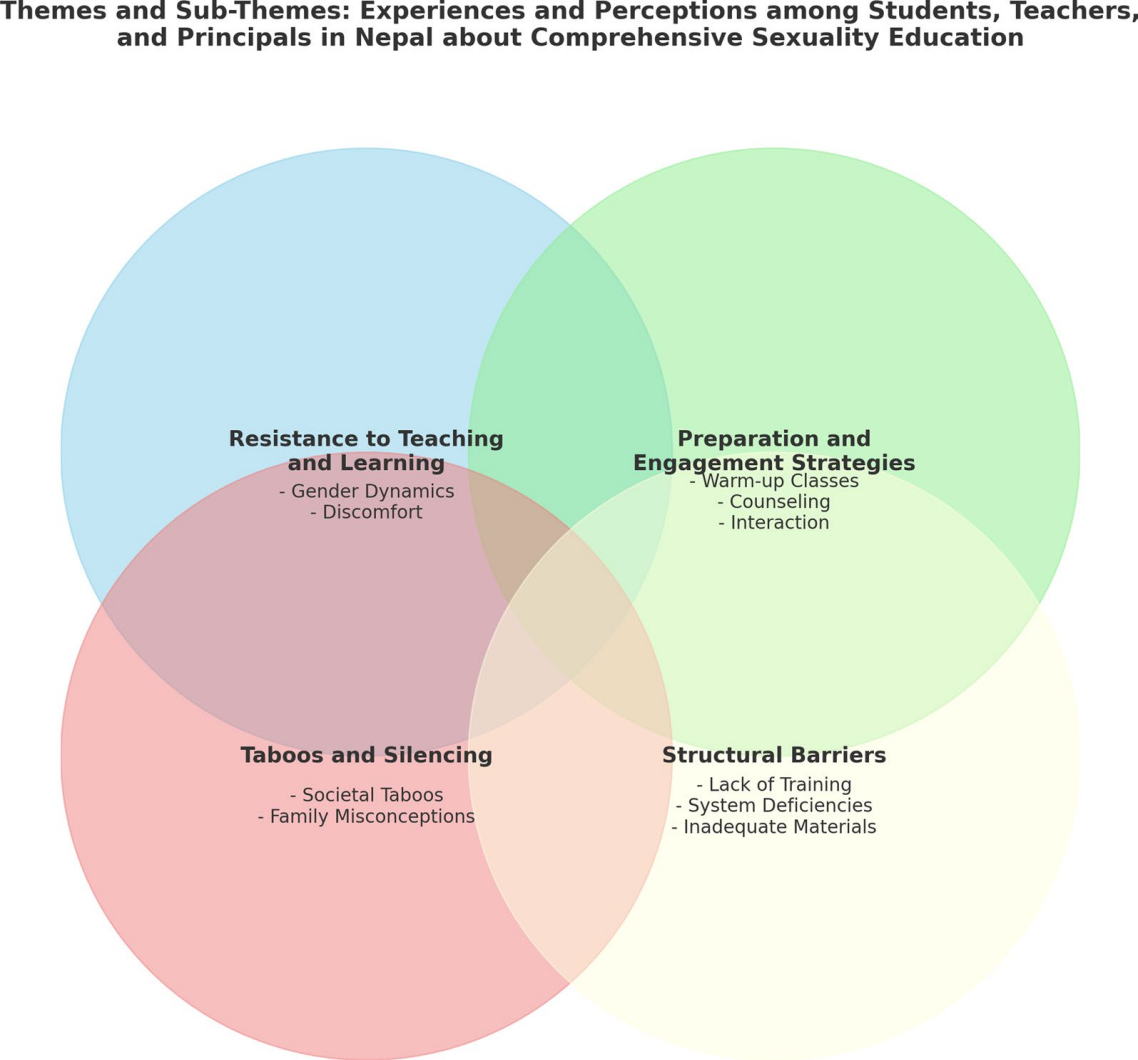
Themes and sub-themes

Four themes and 10 sub-themes were identified: Resistance to Teaching and Learning, Preparation and Engagement Strategies, Taboos and Silencing and Structural Barriers. See Fig. 1 for overview of themes and sub-themes and Table 2 for overview of findings.

**Table 2:** Key findings and supporting verbatims

Theme	Sub-theme	Key findings	Supporting verbatim
Resistance to teaching and learning	Gender dynamics	Teachers and students expressed discomfort due to gender dynamics during CSE classes	"It is easier for me to teach such a topic to boys, but the same does not happen with girls." (Teacher 2)
	Discomfort	Both teachers and students often felt uncomfortable discussing sensitive topics	"We feel like these topics are a bit awkward to discuss in the classroom." (Student 9)
Preparation and engagement strategies	Warm-up classes	Warm-up sessions helped students feel more comfortable and attentive during CSE classes	"Warm-up session should be there before starting CSE… so they can be mentally prepared." (Teacher 10)
	Counseling	Early counseling sessions were critical in preparing students emotionally	"I decided to warm up and discuss their problems with them for two days." (Teacher 2)
	Interaction	Continuous interaction between teachers, students, and parents fostered a trusting environment	"Interaction and discussion with students and parents are the only effective way for such education." (Teacher 4)
Taboos and silencing	Societal taboos	Cultural norms significantly hindered open discussion of CSE topics	"Sex and reproductive health education is not openly discussed in our society now." (Teacher 9)
	Family misconceptions	Family beliefs often led to misconceptions and absenteeism among students	"I have found some cases where students were misguided by their family." (Teacher 12)
Structural barriers	Lack of training	Teachers highlighted the lack of specialized training for CSE	"I have not received formal training for teaching comprehensive sexuality education." (Teacher 15)
	System deficiencies	Inadequate curriculum and materials were significant barriers to effective CSE delivery	"If they develop audio or videos as per requirement, it would be easy for us to teach effectively." (Teacher 3)
	Inadequate materials	The use of outdated or insufficient materials hampered the learning process	"In a government school, there are black and white pictures, and it’s difficult to understand." (Student 4)

## Resistance to teaching and learning comprehensive sexuality education

### Gender dynamics

Teachers and students alike acknowledged the challenges associated with teaching sensitive topics. For teachers, these challenges encompassed the need for expertise and emotional competence, with many emphasizing the significance of gender in dealing with these subjects. Female students found male teachers discussing such topics to be uncomfortable, and a male teacher acknowledged this by stating:

I see one defect: it is easier for me to teach such a topic to boys, but the same does not happen with girls. Maybe female teachers also have the same experiences as mine. (Teacher 2)

Students also had their reservations and discomfort, as one student candidly admitted:

We feel like these topics are a bit awkward to discuss in the classroom. It's not something we're used to, and it can be embarrassing sometimes. (Student 9)

### Teacher and student discomfort

Resistance to teaching and learning comprehensive sexuality education emerged as a significant theme voiced by both teachers and students. Teachers described facing discomfort during CSE classes, often due to students' inappropriate reactions, unrelated questions, or personal inquiries. A teacher revealed:

Sudden student laughter while teaching certain topics of CSE and the sudden rise of random questions from the students can make teachers uncomfortable. (Teacher 3)

However, some teachers adapted over time and no longer considered it a barrier, as one teacher shared:

Previously, I used to feel uncomfortable while teaching classes on those topics, but later on, it did not feel uneasiness to me. (Teacher 12)

Students, too, faced their own set of challenges. They often found themselves feeling uncomfortable during CSE classes. One student explained:

I could sense the facial expression of my classmates while the teacher was teaching such topics. Though they do not say anything, their facial expression says that the teacher is teaching vulgarity. (Student 6)

Nevertheless, over time, students experienced positive changes, becoming more comfortable raising issues related to SRH:

This education has been effective among adolescents. Girls used to feel shy with the fear of getting teased by the boys, but now they come by themselves, asking when the school will distribute free pads. (Student 4)

## Preparation and engagement strategies

### Warm-up sessions and counselling

The teachers interviewed described the methods they used to prepare students mentally and emotionally for the topics included in CSE and to actively engage them in the learning process. These strategies included warm-up classes, early counseling sessions, and continuous interaction with students and parents whose goal was to create a comfortable environment conducive to learning and promote active student participation.

Participants mentioned that the warm-up sessions and explaining the importance of CSE in the daily life of the students made them more attentive in class and less embarrassed when teachers raised topics related to sexual and reproductive health. Participants had described as:

.. warm-up session should be there before starting CSE, just like warm up for starting games so that they can also be mentally prepared for the next classes and feel comfortable. (Teacher 10)

.. I decided to warm up and discuss their problems with them for two days. And I convinced them not to laugh whenever they saw me drawing pictures as it is essential as other organs in our body and important for them to know (Teacher 2)

### Continuous interaction and involvement of parents

The teachers also believed that continuous interaction with the students helped create trust between them. In addition to this, active student participants and also including parents were highlighted as important strategies:

Interaction and discussion with students and parents are the only effective way for such education. Students need to know the facts of different events related to SRH around society. (Teacher 4)

.. the active participation of students can make such education very effective among adolescent students. (Teacher 10)

## Taboos and silencing

### Societal taboos

Teachers acknowledged that societal taboos surrounding sexual and reproductive health topics were pervasive and acted as a significant barrier. A teacher explained:

Sex and reproductive health education is not openly discussed in our society now. Though we teach, students cannot adapt to it because of societal thoughts and judgment. (Teacher 9)

These barriers often resulted from deep-rooted cultural norms that shrouded these subjects in silence. Misconceptions arising from family practices and cultural beliefs also played a pivotal role in hindering the delivery of CSE. Students were frequently misled by their families, leading to absenteeism and misunderstandings. A teacher pointed out:

I have found some cases where students were misguided by their family. I feel like students are victimized because of family culture. (Teacher 12)

### Family misconceptions

This was confirmed by the students who described how family attitudes influenced by societal norms discouraged the adolescents from seeking or receiving comprehensive sexual education:

If we try to share such information to our family also, they will either ignore or demotivate us saying that we are just a child and there is no need to learn more about this. (Student 2)

## Structural barriers

### Educational and administrative challenges

Both teachers, principals and students highlighted structural barriers associated with the educational system and administrative aspects of CSE. These barriers were categorized into executive and educational system deficiencies.

Teachers emphasized the challenges arising from their diverse educational backgrounds and a lack of specialized training. A teacher mentioned seeking help from a science teacher or the internet to enhance their effectiveness in teaching CSE, stating:

I take help from the biology teacher for explaining certain topics so that students understand better and can prevent them from problems. (Teacher 8)

They also pointed out the lack of formal training and professional development opportunities for teaching SRHE topics, with a teacher admitting:

I have not received formal training for teaching comprehensive sexuality education to the students. Also, I have not participated in seminars or meetings regarding such topics. (Teacher 15)

### Deficiencies in curriculum and resources

Educational system deficiencies included issues such as a lack of instructional materials and supplies, an inadequate curriculum, and rigid time schedules. Teachers cited the absence of prescribed videos, charts, and figures as a significant challenge in delivering SRHE, with one teacher expressing:

While developing the curriculum, if they develop audio or videos as per requirement, then it would be easy for us to teach effectively. (Teacher 3)

Furthermore, they pointed out that the curriculum's theoretical approach needed more practical components, such as demonstrations of condom use. A teacher said:

I feel if the topics regarding practical demonstration of condoms were included, then it would have been better because everyone knows condoms should be used for preventing STDs, but how to use is not taught. (Teacher 4)

The students also believed there were structural challenges within educational systems that impeded the delivery of comprehensive sexual education. Participants identified inadequate curriculum and educational materials as significant barriers. They advocated for improvements in the curriculum, including the early introduction of CSE topics and the inclusion of more relevant content:

Whatever contents are presently available are not sufficient, and there should be more education on adolescents’ changes, their problems and also on drug use (Student 8)

According to some participants, the lack of use of modern technology by teachers, such as videos, charts and the use of black and white pictures in textbooks, created difficulty in receiving exact information regarding CSE. One of the participants explained as:

In a government school, there are black and white pictures, and it’s difficult to understand. E.g., we recognize the word pills but can’t identify its real picture as it’s black in color. (Student 4)

## Discussion

The study investigated perceptions and experiences related to sexuality education in Nepal, focusing on barriers to accessing comprehensive sexuality education (CSE) among students, teachers, and principals. Both groups acknowledged significant challenges, including discomfort, embarrassment, and societal judgment, hindering effective teaching, and learning of CSE. Teachers highlighted difficulties in addressing sensitive topics due to students' reactions and societal taboos, while students expressed discomfort and shame influenced by cultural norms. To address these barriers, teachers employed strategies such as warm-up sessions and counseling, aiming to create a supportive learning environment. However, taboos and silencing, including gender role expectation and dynamics, as well as structural barriers within the educational system, such as deficiencies in teacher training and curriculum, further hindered effective delivery of CSE.

One of the primary challenges identified in the current study is the persistence of discomfort surrounding adolescent sexuality in many societies [21, 34, 35]. This discomfort is often rooted in socio-cultural norms and religious beliefs, leading to resistance towards open discussions about sensitive topics related to sexuality. Sexual rights are by some foreign or western concepts, alien to Nepali culture. Previous studies have found an ambivalence towards these rights, viewing them as disruptive to traditional norms and collective identity [35–39]. Such ambivalence is reflected in the reluctance of parents and religious leaders to support CSE, perceiving it as a threat to cultural values [38, 40–43]. Some religious beliefs support communication on sexual and reproductive health (SRH) issues, while others oppose topics deemed culturally inappropriate or immoral [44]. Institutionalized religions and political leaders often opposed CSE implementation, citing concerns about corrupting young people and violating cultural values [20]. One student participating in the current study believed s/he could never raise the topics learnt during CSE at home. It has been found that parents, as crucial stakeholders, influence CSE implementation, with some opposing it due to cultural values and beliefs [37, 40, 45]. Resistance stemmed from discomfort with certain topics and the perception that CSE challenged traditional norms [40, 46, 47] with some considering some of the topics covered to be promoting Western culture over local traditions [39, 44, 48].

This reluctance to engage with comprehensive sexuality education perpetuates gaps in knowledge and skills among adolescents. Teachers expressed varied attitudes towards implementing comprehensive sexuality education (CSE). While some were motivated and appreciated the opportunity to teach adolescents, others hold negative attitudes influenced by cultural and normative factors [49]. Many teachers are more comfortable discussing topics like abstinence and life skills, while sensitive topics such as contraception and homosexuality were often skipped or dropped [40, 46]. One study found that the discomfort was sometimes due to teachers' lack of cultural understanding of their students, leading to hesitation in openly discussing CSE [46]. Gender also played a role, with male teachers finding it difficult to teach certain topics to female students which is in line with a previous study and should be discussed when implementing CSE programs [42]. In some cases, teachers felt that discussing sexuality openly would lead to social stigma, where both they and their students might be labeled as “perverted” or “immoral”, further inhibiting open discussions [42].

In the student's experiences, CSE would be effective when delivered using interactive approaches, emotionally engaging students in discussions about topics like teenage pregnancies and early marriages [23, 45]. Digital and mobile technologies offer innovative solutions for engaging parents and communities in CSE, providing accessible and interactive platforms for communication and education. Digital interventions may be useful in order to overcome barriers such as stigma and cultural taboos, particularly in contexts where face-to-face interactions may be challenging [6, 21]. Interactive and participatory teaching methods have also been shown to be effective in engaging students and promoting understanding of sensitive topics [3, 21, 23, 43, 45]. By incorporating role-play, group discussions, and other interactive activities, teachers can create safe spaces for students to explore and discuss issues related to sexuality openly, as highlighted by the study's findings [49]. However, while these strategies offer some progress, they must be culturally sensitive and appropriate to the local context to ensure their effectiveness.

The study findings highlight the lack of adequate teacher training as a significant barrier to the effective delivery of CSE. Teacher training is crucial for the successful implementation of comprehensive sexuality education (CSE) programs [42, 43, 50]. Critical shortages of human resources in this area have been reported, with some teachers lacking the necessary knowledge and skills to effectively teach CSE [51]. To address this gap, extensive teacher training initiatives have been rolled out at both pre-service and in-service levels [40, 49] decentralized from national to regional, district, and school levels [52]. This approach aims to build capacity among teachers and other stakeholders, fostering a greater sense of ownership and commitment to CSE implementation [48, 52]. Teacher training in CSE has been shown to yield numerous benefits, including increased recognition of the importance of CSE among educators and administrators [40, 53]. Trained CSE teachers are more motivated to teach CSE and better equipped to understand classroom dynamics and handle difficult relationships and cultural issues compared to untrained or under-trained teachers [43]. Moreover, trained teachers are adept at integrating CSE across different subjects [54]. Conversely, the lack of teacher training is identified as a critical issue hindering effective CSE implementation [37, 45]. Untrained teachers often exhibit resistance to CSE due to negative attitudes and challenges in delivering the content [41]. They may also struggle to integrate CSE into their teaching and omit crucial topics, resulting in a decline in the quality of CSE [41, 45, 54]. Addressing these challenges through comprehensive teacher training programs is essential but it should also be accompanied by structural changes that address the sociopolitical, psychosocial and economic barriers that prevent the successful scale-up and integration of CSE into the educational system.

The fact that less than a quarter of Nepalese adolescents have access to CSE indicates large differences in the level of integration of CSE into educational systems across different regions. This is common across low-income countries [22, 34]. While some countries have made significant progress in integrating CSE into formal education systems, others have faced challenges due to factors such as inadequate training materials, poor standardization, and low teacher acceptance [49]. These disparities underscore the need for targeted interventions and support to ensure equitable access to comprehensive sexuality education for all adolescents. However, for these interventions to be truly effective, they must be sensitive to the local context and address deep-rooted taboos and social norms that continue to influence perceptions and implementation of CSE in Nepal. The findings of this study are applicable to educators, policymakers, and community leaders in Nepal, as they provide critical insights into the challenges and opportunities for improving CSE. The results may be used to inform the development of more effective CSE programs that are culturally sensitive and tailored to address the unique socio-cultural and structural barriers identified in Nepalese contexts. The emphasis on continuous interaction with students and parents as a strategy to overcome resistance to CSE, while supported by some literature, might be underexplored in the context of Nepal. Further research should aim to evaluate the effectiveness of such strategies in overcoming socio-cultural barriers in CSE delivery. Additionally, research should investigate the effectiveness of digital and community-based interventions in overcoming barriers to CSE, including societal taboos and limited access in rural areas.

### Study limitations

While the study provides valuable insights into the barriers and challenges associated with the implementation of CSE in Nepal, it also has some limitations. The sensitive nature of the topic could have led to underreporting or reluctance to fully disclose personal experiences, especially among students who might fear judgment or repercussions. The selection of students for focus group discussions (FGDs) was done by their teachers, which may introduce selection bias. Teachers might have chosen students who they believed would provide more favorable or articulate responses, thus not fully capturing the diversity of student experiences and opinions. Another limitation of this study is the decision not to report the sex of the teacher participants due to the small sample size, which could have compromised their anonymity. As a result, the potential impact of gender dynamics on the teachers' narratives and experiences with CSE was not explicitly explored. As a qualitative study, the sample is not representative of the broader population, as it was limited to specific schools and regions in Nepal. This geographic and institutional focus may have excluded variations in experiences and perceptions that could exist in other parts of the country. Lastly, the study was conducted in Nepali and later transcribed and analyzed. While every effort was made to ensure accurate translation and interpretation, nuances and contextual meanings might have been lost or altered during this process.

## Conclusion

Investigating the experiences and perceptions among students, teachers, and principals in Nepal about comprehensive sexuality education, the study findings highlighted both socio-cultural and structural barriers. Teachers and students frequently expressed discomfort and embarrassment when discussing sensitive topics, a situation exacerbated by gender role expectation and dynamics as well as societal norms. Additionally, structural barriers such as inadequate teacher training, lack of instructional materials, and insufficient curriculum content compounded these issues. Despite these obstacles, the study identified positive changes and opportunities. Some teachers and students reported growing comfortable with CSE over time. Strategies such as warm-up sessions and continuous interaction with students helped foster a supportive learning environment. Addressing the challenges identified in the study requires comprehensive teacher training, curriculum reform, and community engagement to establish a supportive environment for adolescents. Overcoming the deeply ingrained norms and power structures that resist CSE necessitates a multifaceted approach, including broader societal engagement, policy reform, and efforts to shift cultural perceptions. Ensuring equitable access to CSE across all regions of the country is essential for empowering young people to manage their sexual and reproductive health effectively. By leveraging these positive strategies and opportunities, it is possible to enhance the delivery and effectiveness of CSE in Nepal.

## Supplementary Information

**Additional file 1. **Interview guides.

## Data Availability

No datasets were generated or analysed during the current study.
