# Supplementary material for: Exploring comprehensive sexuality education experiences and barriers among students, teachers and principals in Nepal: a qualitative study
Source: Reprod Health. 2024 Sep 11;21:131. doi: 10.1186/s12978-024-01876-0 (PMC11389116; doi:10.1186/s12978-024-01876-0)
Supplement: Supplementary file 1 — Additional file 1. Interview guides. [file 12978_2024_1876_MOESM1_ESM.pdf]

Interview Guides (English and Nepali)

Interview Guide of Subject Teachers

Background information

- How long have you been in the teaching field?
- How long have you been teaching grade 9 and grade 10?
- How many working places have you changed until now?
- Have you ever encountered the dropout of students because of marriage, pregnancy, or any other causes in the last few years?

Past Experiences

- How was your experience of receiving sexual reproductive knowledge during your adolescent age? *How did teachers approach students? were all your queries answered? How did you approach teachers?*
- What do you feel about those teaching experiences? *Do those teaching experiences influence you? How?*

Present Experience

- Have you ever resisted teaching this reproductive health information by cultural norms and religious beliefs? How?
- How are classes organized in school? *Is there any specific school protocol for the classes on sexual and reproductive health knowledge? what arrangements are priory prepared before classes, and how are adolescent students' different reactions handled during these classes? How do you act in those situations?*
- What approach do you apply while teaching issues on sexual reproductive health in school? *How did you develop the approach + Do you think it works well? How and why not? How do you feel when using this approach?*

Barriers

- How do you define barriers to facilitating good reproductive health education or information?
- What do you think for the overcome of the barriers to adolescents' reproductive health knowledge? *How do you define your role in addressing the stigma and taboo under menstruation?*

- How do you define the role of educational sectors in the reproductive health knowledge of adolescents? *How can reproductive health knowledge be organized within or outside the school curriculum?*

Interview Guide of Principals

Background information

- How long have you been working as the principal of the school?
- How many working places have you changed until now?
- Have you ever encountered the drop out of students because of marriage, pregnancy, or any other causes in the last few years?

Experiences

- How is reproductive health information delivered in your school settings? *is there any interaction with parents and students about the importance of such education? How or why not?*
- What are your experiences regarding the effect of including reproductive health information in the school curriculum? *how did the teachers and students react to the need for special training for teaching such subjects? what do you think that reproductive health information should be included in the school curriculum from grade 8 or lower grades? If yes, how and if no, why?*

Barriers and future perspectives

- What do you think about overcoming the barriers to adolescents' reproductive health knowledge? *Have you initiated awareness programs on the importance of sexual and reproductive health knowledge at the school or in society? if yes, how and if no, why?*
- What do you think about the role of educational sectors in the reproductive health knowledge of adolescents? *what do you think about organizing the course content of reproductive health education broadly within the school educational system or not?*

FGD Guide of Subject Teachers

Inclusion of reproductive and sexual health education (SRHE) in the school curriculum

- How do you feel about including reproductive and sexual health education in the school curriculum? *Are all the necessary topics covered in the curriculum? Do you think any content is not to be included or to be added to the curriculum for adolescents? Why and why not?*

  An effective way to deliver SRHE
- What do you think about how sexual and reproductive knowledge can be delivered to adolescents effectively from school? *Is it applicable, durable and how? Have you tried it anyways and found it effective? How to assess their effectiveness?*

  Barriers
- What are the main barriers towards adolescent reproductive and sexual health education in Nepal?
- Which one among several barriers has restricted you from delivering effective education, why and how?
- What do you think for the overcome of the barriers towards adolescents' reproductive and sexual health education? *Why and how?*

अन्तर्वार्ता सम्बन्धि विवरण

पृष्ठभूमि

- तपाईं अध्यापन पेशामा कति लामो समय देखि आवद्ध हुनुहुन्छ?

-तपाईं कक्षा ९ र कक्षा १० को शिक्षकका रुपमा कहिले देखि कार्यरत हुनुहुन्छ?

-तपाईंले अहिले सम्म कतिवटा विद्यालयहरुमा पढाउनु भएको छ?

-गएका वर्षहरुमा विवाह, गर्भवती, वा अन्य कुनै कारणले विद्यालय छोडेका विधार्थीहरु बारे तपाईं जानकार हुनहुन्छ?

यस अघिको अनुभव

-तपाईंले आफ्नो किशोरावस्थामा यौन तथा प्रजनन स्वास्थ्य सम्बन्धि शिक्षा प्राप्त गरेको सम्झना छ? *त्यतिखेर शिक्षकहरुले विद्यार्थीसंग कसरि सो विषयमा कक्षामा प्रस्तुत हुन्थे? तपाईंका ती बेलाका सबै जिज्ञासाहरु समाधान भएका थिए? तपाईंले शिक्षकसंग आफ्नो जिज्ञासा कसरि राख्नु हुन्थ्यो?*

-त्यसबेलाको शिक्षण विधि बारे अहिले तपाईं कस्तो महसुस गर्नु हुन्छ? *त्यति बेलाको शिक्षण विधि अहिले तपाईंका निम्ति कति प्रेरक भएको छ?*

वर्तमान अनुभव

-तपाईंसंग जोडिएको संस्कृति र परम्पराका कारण यौन तथा प्रजनन स्वास्थ्य सम्बन्धि शिक्षण गर्न नकारेको कुनै अनुभव छ? छ भने तपाईंको अनुभव बताईदिनु होस्।

-तपाईंको विध्यालयमा कक्षाहरु कसरी व्यवस्थापन गरिन्छन? *यौन तथा प्रजनन स्वास्थ्य शिक्षणका निमित्त कुनै छुट्टै मापदण्ड बनाइएको छ? सो विषयको कक्षा लिनु पुर्व कुनै तयारी गरिन्छ? कक्षामा किशोर-किशोरीहरुको जिज्ञासा कसरि समाधान गर्नु हुन्छ?*

-यौन तथा प्रजनन स्वास्थ्य सम्बन्धि विषय पढाउँदा तपाईं कुन शिक्षण विधि प्रयोग गर्नु हुन्छ? *तपाईंले सो विधि समन्धी शिक्षा कसरि हाँसिल गर्नु भयो? तपाईंले हाल प्रयोग गरिरहेको शिक्षण विधिले प्रभावकारी भए जस्तो लागछ?*

शिक्षणका कठिनाईहरु

-यौन तथा प्रजनन स्वास्थ्य सम्बन्धि शिक्षणबाट किशोर-किशोरीहरुको चेतना अभिवृद्धिमा उत्कृष्ट नतिजा हाँसिल गर्न के-कस्ता कठिनाईहरु महसुस गर्नु भएको छ?

-तपाईंले ती कठिनाइहरु कसरी समाधान गर्नु भएको छ? *रजस्वला सम्बन्धमा रहेका गलत बुझाइ र सोचहरुमा परिवर्तन ल्याउन कस्तो उपाय अवलम्बन गर्नु भएको छ?*

-किशोर-किशोरीहरुका निम्ति यौन तथा प्रजनन स्वास्थ्य सम्बन्धि शिक्षा प्रदान गर्न शैक्षिक संस्थाहरुमा हुने शिक्षण कत्तिको प्रभावकारी छ? *यौन तथा प्रजनन स्वास्थ्य समबन्धि चेतना अभिवृद्धि गर्न विद्यालय भित्र र वाहिरको अभ्यासलाइ अझ कसरी परिष्कृत र प्रभावकारी बनाउन सकिएला ?*

अन्तर्वार्ता सम्बन्धि विवरण

पृष्ठभूमि

- तपाईं यस विद्यालयमा कहिले देखि प्रधानाध्यापक हुनुहुन्छ?

-तपाईंले अहिले सम्म कतिवटा विद्यालयहरुमा काम गर्नु भएको छ?

-गएका वर्षहरुमा विवाह, गर्भवती, वा अन्य कुनै कारणले विद्यालय छोडेका विधार्थीहरु बारे तपाईं जानकार हुनहुन्छ?

यस अघिको अनुभव

-तपाईं कार्यरत विद्यालयमा प्रजनन स्वास्थ्य सम्बन्धि शिक्षा प्रदान गर्ने कुनै निश्चित विधि विकास गरिएको छ? *सो शिक्षाको महत्व बारे अविभावक र विद्यार्थीहरुसंग कुनै छलफल गर्नु भएको छ ?*

-यौन तथा प्रजनन स्वास्थ्य शिक्षा पाठ्यक्रममा समावेश गर्दाको प्रभाव बारे तपाईंको अनुभव कस्तो छ? *सो विषयको शिक्षणका लागि शिक्षक तथा विद्यार्थीहरुलाई विशेष तालिम प्रदान गर्न कुनै प्रयास गर्नु भएको छ ? कक्षा ८ तथा तल्ला कक्षाहरुको पाठ्यक्रममा यौन तथा प्रजनन स्वास्थ्य शिक्षा समावेश गर्न उपयुक्त ठान्नुहुन्छ ? हो भने किन, र होइन भने किन ?*

शिक्षणका कठिनाईहरु

-यौन तथा प्रजनन स्वास्थ्य सम्बन्धि शिक्षणबाट किशोर-किशोरीहरुको चेतना अभिवृद्धिमा उत्कृष्ट नतिजा हाँसिल गर्न के-कस्ता कठिनाईहरु महसुस गर्नु भएको छ? *यौन तथा प्रजनन स्वास्थ्य शिक्षाको महत्व बारे विद्यालय तथा सामुदायिक स्तरमा सचेतना सम्बन्धि कुनै प्रयास गर्नु भएको छ ?*

-किशोर-किशोरीहरुका निम्ति यौन तथा प्रजनन स्वास्थ्य सम्बन्धि शिक्षा प्रदान गर्न शैक्षिक संस्थाहरुमा हुने शिक्षणको भूमिका कस्तो छ? *विद्यालयको शिक्षण गतिविधि अन्तर्गत यौन तथा प्रजनन स्वास्थ्य शिक्षा सम्बन्धि पाठ्यवस्तु कसरी व्यवस्थापन गर्नु हुन्छ ?*

Interview Guide- students

Inclusion of reproductive and sexual health education (SRHE) in school curriculum

- How do you feel about the inclusion of reproductive and sexual health education in school curriculum? *Are all the necessary topics covered in curriculum? Do you think any contents not to be included or to be added in the curriculum for the adolescents, why and why not?*

Effective way to receive SRHE

- Whom do you share your problems related to SRH such as physical and emotional changes during adolescents, hormonal changes, sexual protection and so on with? Are they family members, friends, schoolteachers or other? *If any, why? How and what sorts of problems are commonly discussed or shared with them?*
- What do you think on the roles of parents for SRHE? *Whom (family members) do you share your problems with and how?*
- What do you think how sexual and reproductive knowledge can be delivered to adolescents in effective way from school? *How have you been taught till now and has it met your expectations?*
- What do you think how the students can ask any sort of questions frankly to teachers regarding SRHE? Any new techniques or suggestions for school or schoolteachers.

Barriers

- What are the main barriers towards adolescent´s reproductive and sexual health education in Nepal?
- Which one among several barriers has restricted you in receiving effective education, why and how?
- What do you think for the overcome of the barriers towards adolescent's reproductive and sexual health education? *Why and how?*

अन्तर्वार्ता सम्बन्धि विवरण

- **विद्यालय शिक्षा पाठ्यक्रममा यौन तथा प्रजनन स्वास्थ्य शिक्षाको समायोजन**

-पाठ्यक्रममा यौन तथा प्रजनन स्वास्थ्य शिक्षा समावेश गर्नु पर्ने विषयमा तपाईंको धारणा कस्तो छ? *के सबै विषयहरु पाठ्यक्रममा समावेश छ? पाठ्यक्रममा समावेश गर्न नमिल्ने कुनै त्यस्ता यौन तथा प्रजनन सम्बन्धि विषयहरु पनि छन् ? ती विषय किन समावेश गर्न मिल्दैन?*

- **यौन तथा प्रजनन स्वास्थ्य शिक्षा प्रदान गर्ने उत्तम उपाय**

-तपाईंको विचारमा प्रभावकारी ढंगले यौन तथा प्रजनन स्वास्थ्य शिक्षा प्रदान गर्ने उपायहरु के के हुन सक्छन? *तपाईंले प्रयोग गर्ने गरेको शिक्षण विधि कत्तिको प्रभावकारी पाउनु भएको छ ?*

- **शिक्षणका कठिनाईहरु**

-यौन तथा प्रजनन स्वास्थ्य सम्बन्धि शिक्षणबाट किशोर-किशोरीहरुको चेतना अभिवृद्धिमा उत्कृष्ट नतिजा हाँसिल गर्न के-कस्ता कठिनाईहरु महसुस गर्नु भएको छ?

-तपाईंले महशुस गर्नु भएका अप्ठेराहरु मध्य कुनले शिक्षा प्रदान गर्न कठिन बनाएको छ? किन र कसरी?

-तपाईंले ती कठिनाइहरु समाधान गर्न केही सोच्नु भएको छ? *किन र कसरी ?*
